# Enhancing biomass and lipid productivity of a green microalga *Parachlorella kessleri* for biodiesel production using rapid mutation of atmospheric and room temperature plasma

**DOI:** 10.1186/s13068-022-02220-z

**Published:** 2022-11-13

**Authors:** Mostafa E. Elshobary, Hossain M. Zabed, Xianghui Qi, Rania A. El-Shenody

**Affiliations:** 1 Department of Botany, Faculty of Science, Tanta University, Tanta, 31527 Egypt; 2 School of Food and Biological Engineering, Jiangsu University, Zhenjiang, 212013 Jiangsu China

**Keywords:** Atmospheric and room temperature plasma, *Parachlorella kessleri*, Lipid production, Biodiesel quality, Fatty acid, Triglyceride

## Abstract

**Background:**

Microalgae, with their high adaptability to various stress conditions and rapid growth, are considered excellent biomass resources for lipid production and biodiesel feedstocks. However, lipid yield and productivity of the natural strains are common bottlenecks in their large-scale use for lipid production, which can be overcome by evolving new strains using conventional and advanced mutagenic techniques. It is challenging to generate microalgae strains capable of high lipid synthesis through natural selection. As a result, random mutagenesis is currently considered a viable option in many scenarios. The objective of this study was to explore atmospheric and room temperature plasma (ARTP) as a random mutagenesis technique to obtain high lipid-accumulating mutants of a green microalga for improved biodiesel production.

**Results:**

A green microalgal species was isolated from the Chinese Yellow Sea and identified as *Parachlorella kessleri* (OM758328). The isolated microalga was subsequently mutated by ARTP to obtain high lipid-accumulating mutants. Based on the growth rate and lipid content, 5 mutants (named M1, M2, M4, M5, and M8) were selected from 15 pre-selected mutants. These five mutants varied in their growth rate from 0.33 to 0.68 day^−1^, with the lipid content varying between 0.25 g/L in M2 to 0.30 g/L in M8 at 10th day of cultivation. Among the mutants, M8 showed the maximum biomass productivity (0.046 g/L/day) and lipid productivity (20.19 mg/L/day), which were 75% and 44% higher than the wild strain, respectively. The triglyceride (TAG) content of M8 was found to be 0.56 g/L at 16th day of cultivation, which was 1.77-fold higher than that of the wild strain. Furthermore, M8 had the highest saturated fatty acids (C16-18) with the lowermost polyunsaturated fatty acid content, which are favorable properties of a biodiesel feedstock according to international standards.

**Conclusion:**

The mutant strain of *P. kessleri* developed by the ARTP technique exhibited significant improvements in biomass productivity, lipid content, and biodiesel quality. Therefore, the biomass of this mutant microalga could be a potential feedstock for biodiesel production.

**Supplementary Information:**

The online version contains supplementary material available at 10.1186/s13068-022-02220-z.

## Background

Today, fossil fuels (e.g., diesel, coal, and gas) account for 90% of global energy consumption, while the remaining 10% comes from renewable energy sources [1, 2]. The overconsumption of fossil fuels results in the rapid depletion of oil reservoirs, and burning these conventional fuels causes several environmental issues, primarily greenhouse gas (GHG) emissions. As a result, alternative energy sources have received special attention in recent years to solve the energy crisis and reduce GHG emissions. Despite various biomass resources that could be transformed into liquefied fuels, referred to as “biofuels” [3], microalgal biomass is considered to be one of the sustainable alternatives to traditional biodiesel feedstocks [4, 5] owing to their high growth rate, lipid production, and adaptability in harsh environments. Moreover, microalgae do not require arable land for cultivation and can be grown in seawater or wastewater, making them attractive feedstocks for 3rd and 4th generation of biodiesel production [4, 6, 7]. Several studies investigated that the growth of microalgae in different wastewater showed a considerable increase in algal biomass and lipid content compared to control, including vegetable waste extract [7], palm oil mill effluent [8] and industrial wastewater [9].

In order to be able to use microalgae for biodiesel production, they must have a high lipid yield and a high growth rate. Significant lipid synthesis can result when cellular mechanisms shift metabolic fluxes towards lipid accumulation metabolism in response to environmental stress [10, 11]. Even though microalgae can adapt to various stress conditions and grow fast, natural selection is challenging to acquire positive microalgal species for improved lipid synthesis due to their low mutation rate [12]. As a result, mutating microalgal species for high biomass and lipid productivities is a promising tool for biodiesel production. Several mutation procedures were investigated to increase mutation rates and select favorable variants [13]. Genetic engineering appears to be the most effective mutation strategy, but at the same time, it is also the most costly and complex approach. Chemical and physical mutagenesis are also routinely used to increase diversity in microorganisms [14], which include ultraviolet light (UV), γ rays, X-rays, neutrons, β and α particles radiations. However, chemical mutagens are dangerous as their exposure to the users causes high toxicity and necessitates expensive equipment, particular knowledge, and assessment [15].

**Fig. 1:**
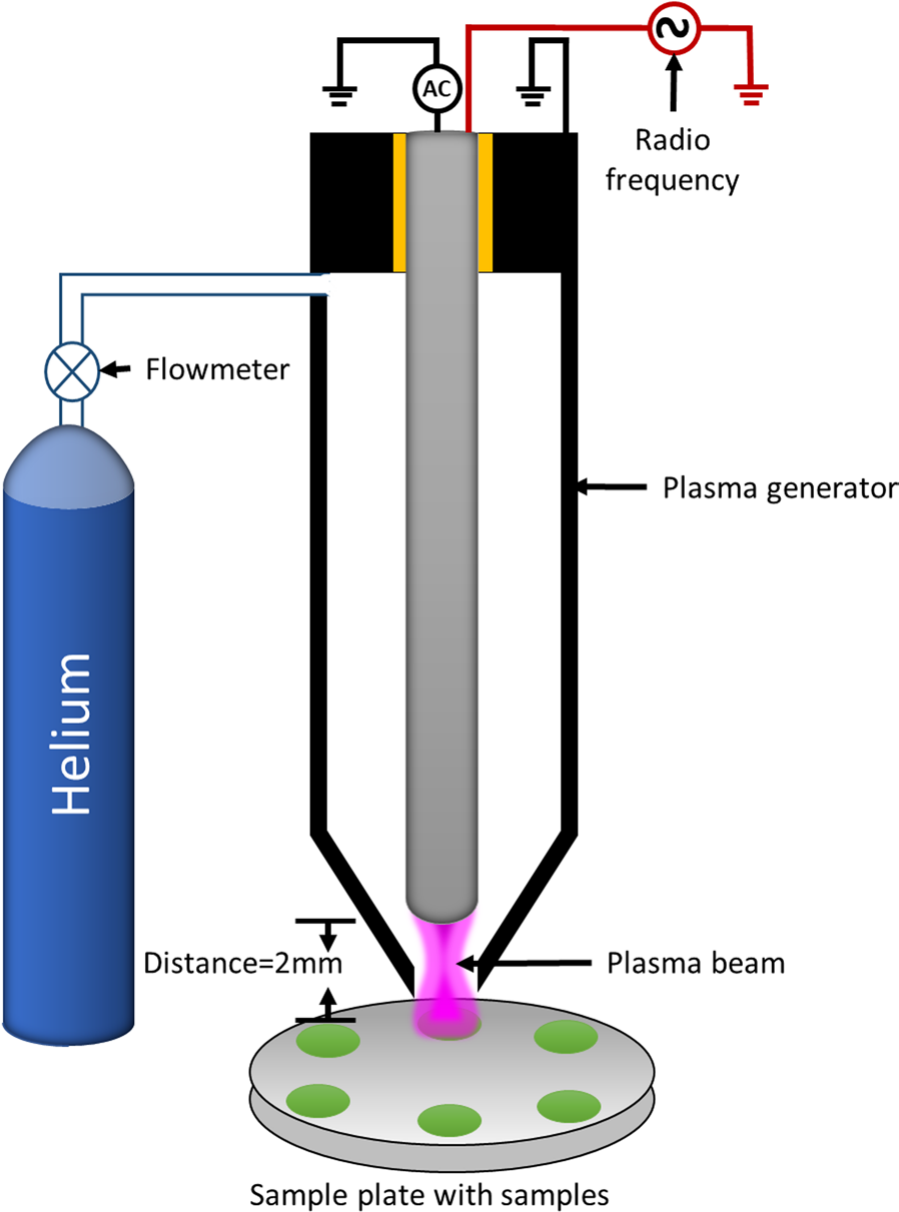
Scheme of ARTP mutagenesis for microbial mutation

In contrast, atmospheric and room temperature plasma (ARTP) is a powerful, rapid, effective, low-cost and eco-friendly physical mutagenesis system that leads to biodiversity in the mutants. ARTP depends on high-purity helium flow in a high-frequency electric field at low temperatures [16] (Fig. 1). ARTP causes considerably more genetic damage than UV, nitroquinoline oxide and diethyl sulfate. Moreover, its mutation rate was significantly higher, increasing the chances of acquiring promising mutants [17]. During the mutation process, ARTP could substantially alter the physicochemical characteristics of the cell wall and cell membrane by lessening the surface potential, causing tissue damage, forcing cells to initiate the Save Our Soul (SOS) repair mechanism with a high capacitance tolerance level, leading to various of interference sites in the repair process. Eventually, the mutant strains' genetic consistency could be accomplished [16]. ARTP was compared with three different mutagenesis systems: ultraviolet radiation, *N*-methyl-*N*′-nitro-*N*-nitrosoguanidine mutagenesis and 4-nitroquinoline-1-oxide. The DNA damage-induced SOS response and mutation rate were inversely correlated. Compared to other traditional mutagenesis techniques, ARTP results in an approximately twofold increase in mutation rate and DNA damage to a single living cell of *Salmonella typhimurium* NM2009 [17].

ARTP has been successfully used to mutate various microorganisms, including bacteria, fungi, and microalgae [12, 18]. For example, a mutant microalga, C*hlorella pyrenoidosa,* was developed by exposure to ARTP, which improved dry biomass weight and lipid production by 22.07% and 16.85%, respectively, over the original strain [16]. Likewise, ARTP was an effective tool for mutating *Chlamydomonas reinhardtii,* which increased hydrogen production over original strains by reducing chlorophyll size [19]. ARTP mutagenesis technique was also reported to increase growth rate and lipid accumulation in a dinoflagellate, *Crypthecodinium cohnii* [16]. There was also an improvement in growth rate, carbohydrate and chlorophyll contents, as well as CO_2_ fixation rate in three mutants of *Spirulina platensis* [20].

The application of the ARTP mutagenesis technique to mutate microorganisms has been confirmed to be feasible due to its high mutagenic rate. Nevertheless, evidence of its use to mutate microalgae for high lipid accumulation is still limited. Furthermore, the use of ARTP in mutating oleaginous marine microalgae, particularly *Parachlorella kessleri* (formerly known as *Chlorella kessleri*), has yet to be reported. *P. kessleri* is a unicellular microalga that belongs to the class Trebouxiophyceae and the family Chlorellaceae, ubiquitous in fresh or marine water. This alga is an excellent candidate for preliminary investigations of the effects of mutagenesis generated by ARTP due to its rapid growth rates and haploid life cycle. Furthermore, it is commercially significant for biomass and lipid production and can be used in bioremediation. Different strategies have been used to improve biomass and lipid production by *Parachlorella* sp., such as the deprivation of macronutrients (N, S, and P) [21, 22] and salt stress [23]. However, the improvement in lipid production has not yet been at a satisfactory level. Moreover, enhancements in lipid production using transgenic microalgae are hindered by the long-term process of transformation practices for these microalgae [24].

In this study, *P. kessleri* was mutated by ARTP under various radiation times to obtain mutant strains with high growth rates and lipid productivities. The selected strains were further examined after five generations and assessed for biomass and lipid production. Comparative estimation of cellular biomass, intracellular component content, triglyceride content, fatty acid profile, and biodiesel properties were assessed.

## Results and discussion

### Phylogenetic identification by 18S rDNA sequencing

**Fig. 2:**
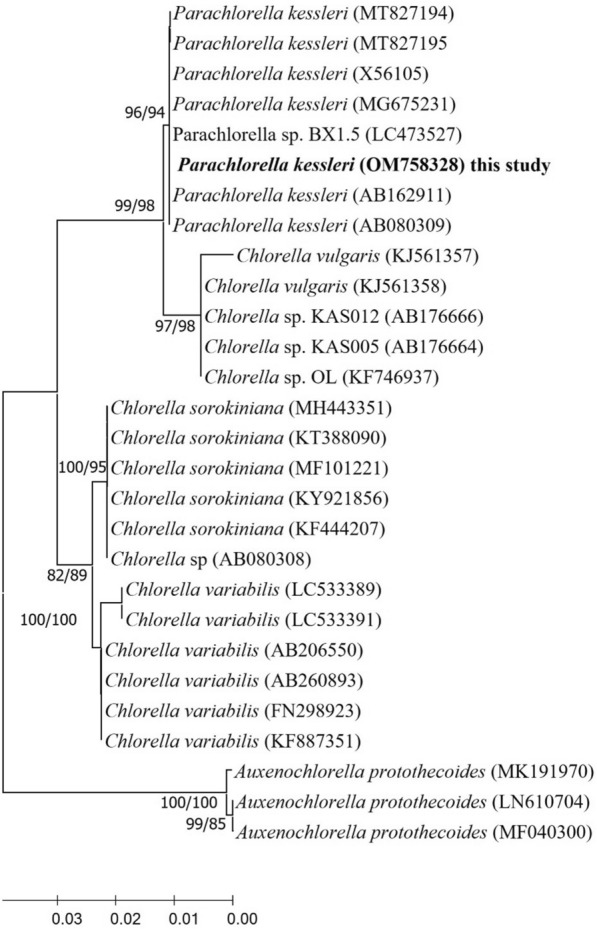
Maximum parsimony (MP) phylogenetic tree for *Parachlorella kessleri* (accession number OM758328) uses constructed 18S rRNA sequences. Bootstrap values greater than 70 are shown on the tree in order of maximum parsimony bootstrap (left) and neighbor-joining (NJ) (right)

The phylogeny of the 18S rDNA gene was derived from approximately 530-bp nucleotide sequences (PCR-based) of the isolated *Parachlorella kessleri*. The maximum parsimony and neighbor-joining phylogeny produced the same topology and comparable bootstrap support values. The 18S gene was aligned with 18S nucleotide sequences from 28 Chlorellales strains in the NCBI rDNA sequence in addition to three sequences of *Auxenochlorella protothecoides* as an outgroup. All ambiguous positions were eliminated using the pairwise removal option for each pair of sequences. Phylogeny analysis produced maximum parsimony (MP) tree with a length of 52 changes, a consistency index (CI) of 0.918, a retention index (RI) of 0.983 and a rescaled consistency index (RC) of 0.903. There was a total of 546 positions in the final dataset of the phylogeny. Evolutionary analyses were accomplished in MEGA X [25]. Each species produced monophyletic clades, and the isolated species was clustered into *Parachlorella kessleri*, achieving a high similarity of 100% and a bootstrap of 94% (Fig. 2). The newly isolated *P. kessleri* strain sequence was placed into the GenBank database (accession number OM758328).

### Growth curve and intracellular composition

**Fig. 3:**
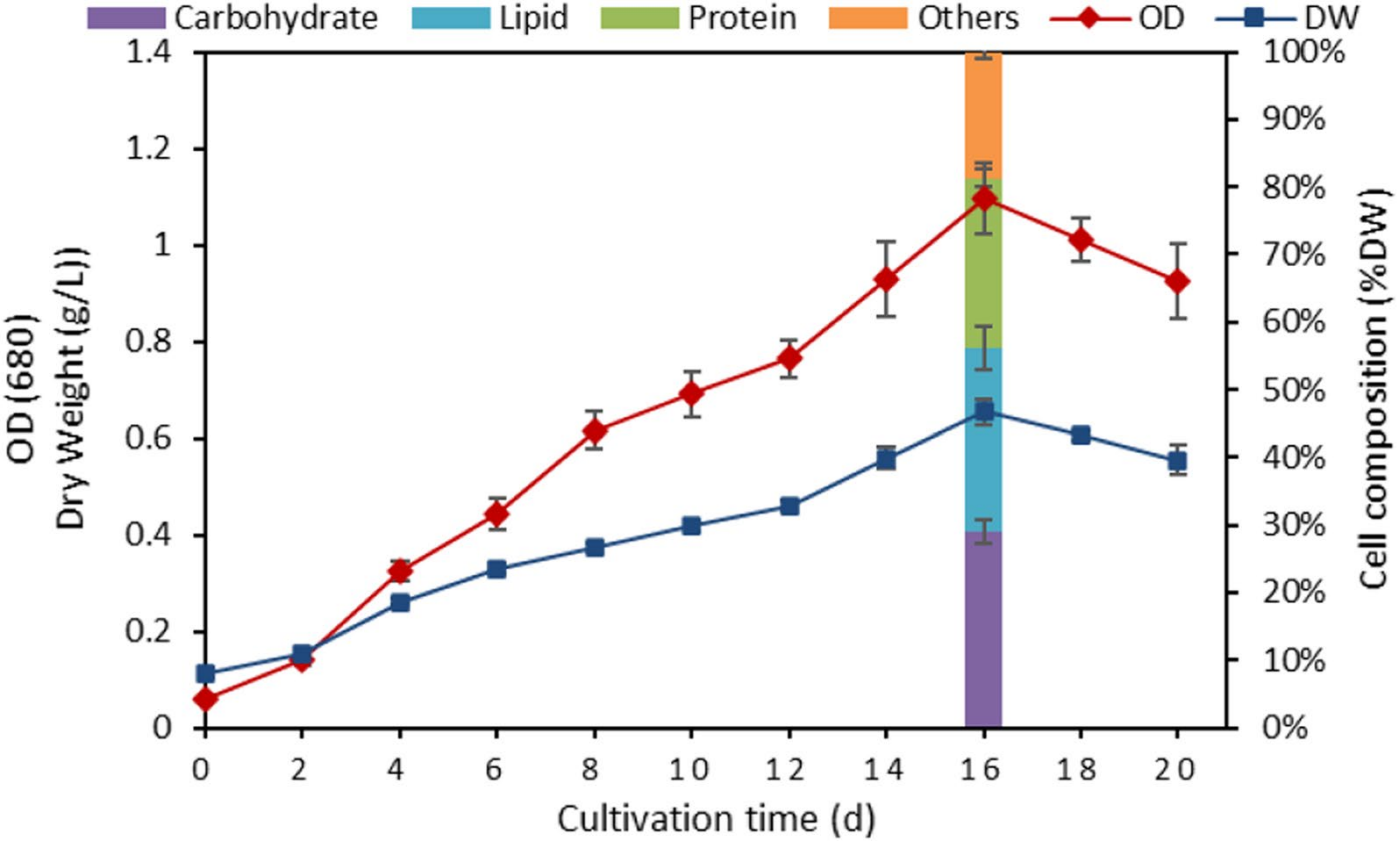
Growth curve of *Parachlorella kessleri* grown at F/2 medium for 20 days demonstrating the primary cellular composition at late log growth phase (16th day)

In a preliminary experiment, the growth phases and cellular composition of *P. kessleri* were evaluated (Fig. 3). It was grown in F/2 medium and incubated until it reached the stationary growth phase. Both OD and dry weight curves showed the same pattern, where the alga had a 2-day lag phase, followed by 14 days of log growth before entering the stationary phase on the 16th day when the maximum OD and dry weight were 1.09 and 0.26 g/L, respectively (Fig. 3). On the 16th day (end of the exponential phase), the contents of carbohydrate, lipid, and protein in microalgal cells were found to be 26.12, 24.43, and 22.66% dry weight (DW), respectively, with biomass of 0.654 g/L (Fig. 3). The biomass of *P. kessleri* in this study was greater than that exhibited by some marine microalgae, such as *Nannochloropsis oculata* (0.234 g/L) and *Chlorella salina* (0. 124 g/L) [26]. On the other hand, biomass was lower than that reported from freshwater microalgae, such as *Arthrospira platensis NIOF17* (0.845 g/L) [27] and *Micractinium reisseri* (1.28 g/L) [11]. The biomass of the present study was within the range reported for the marine *P. kessleri* (0.05–0.54 g/L) but less than that reported for the freshwater species (0.76–0.76 g/L) (Table 1). Therefore, the biomass and lipid productivities of this strain needed to be further improved.

**Table 1:** Comparison of biomass, biomass productivity and lipid content of *Parachlorella kessleri* with related studies reported in the literature

Species	Culture medium	Biomass (g/L)	Biomass productivity (g/L/d)	Lipid content (%)	References
Freshwater *P. kessleri*	Kessler	0.77	0.065	25%	[28]
	BG-11	0.76	0.083	20.9	[29]
Marine water *P. kessleri*	Artificial seawater	0.20	–	31%	[30]
	F/2	0.54	–	35%	[31]
	F/2	0.65	0.035	24.43%	This study

–, means not detected.

### Selection of mutant strains by ARTP

**Fig. 4:**
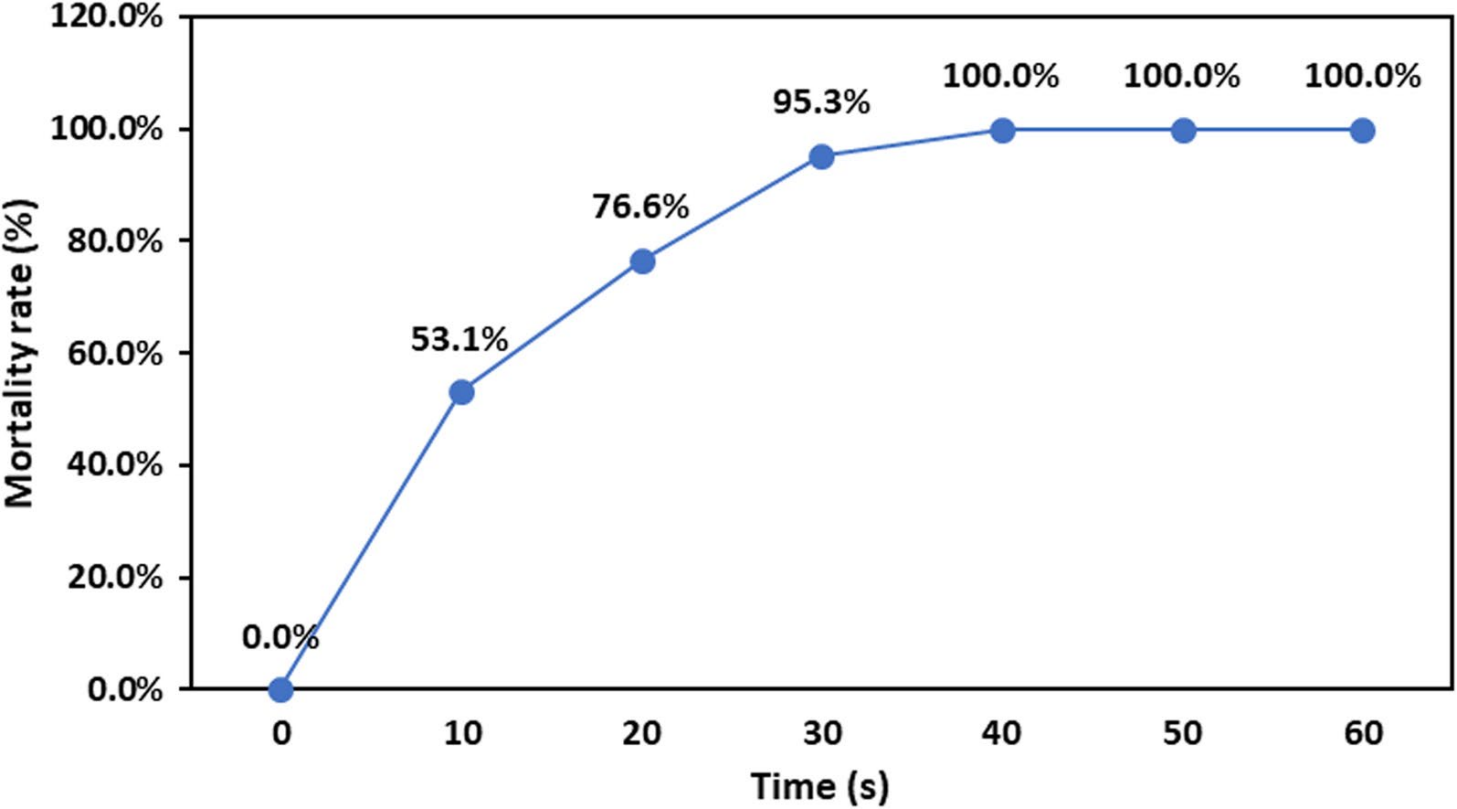
Effects of ARTP exposure time on the mortality rate of *Parachlorella kessleri*

To improve biomass and lipid production, *P. kessleri* was exposed to ARTP for 0 s, 10 s, 20 s, 30 s, 40 s, 50 s and 60 s. The mortality rate (%) of algal cells increased in a sigmoid pattern with an increase in the exposure time of ARTP. As shown in Fig. 4, the mortality rate was 95.3% at 40 s exposure time, which represents 15 colonies. The lethality of cells reached 100% as the treatment time exceeded 40 s. According to the modern mutation principle, the positive mutation rate can be the highest when the mortality rate of cells is ≥95% [12, 16]. To maximize the potential for achieving the desired algal strains with superior lipid production and necessary survivability, a mutagenesis time of 40 s was chosen as the optimum for the ARTP treatment.

**Fig. 5:**
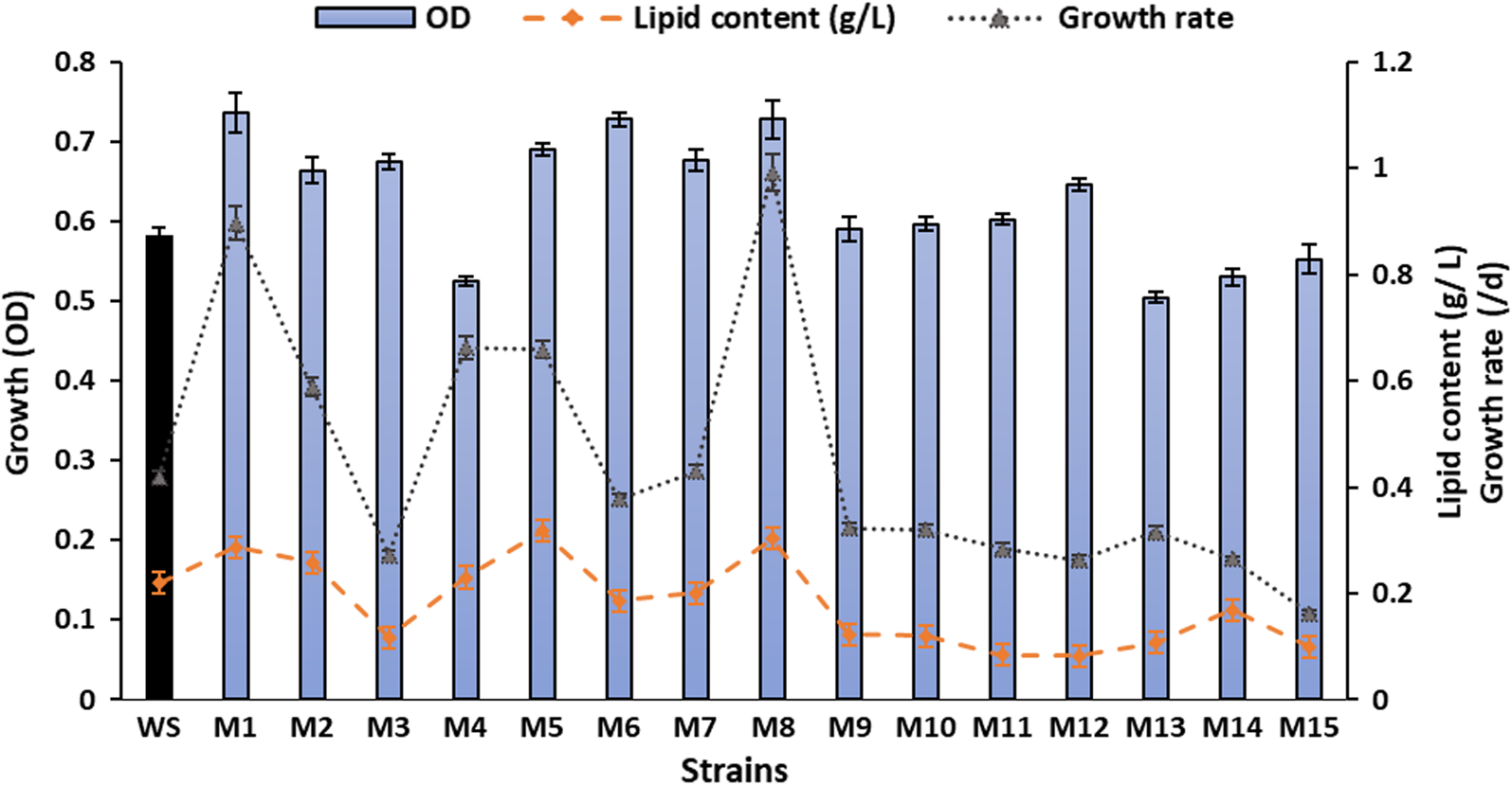
Screening growth (OD) and lipid content (g/L) of the 15 mutant strains of *Parachlorella kessleri* compared to the wild strain after 10 days of growth (wild strain was colored black)

The 15 mutant strains (M1–M15) were picked up and cultivated separately in 50 mL test tubes containing 30 mL of F/2 medium and then incubated in a light incubator with shaking 1–3 times daily. The absorbance of OD was measured after 10 days of growth. After the comprehensive analysis, five mutants, namely M1, M2, M4, M5 and M8, were selected based on their higher cell density, growth rate and lipid content than those of the wild strain (WS). The growth rates of the selected 5 mutants varied from 0.33 day^−1^ in M2 to 0.68 day^−1^ in M8, with 1.6 to 3.44-fold increase from the growth rate of the wild strain. While the lipid content ranged between 0.25 g/L in M2 to 0.30 g/L in M8, with an increase of 1.04 to 1.37-fold compared to the control (Fig. 5). The stabilities of the selected mutants were studied by inoculating in F/2 medium and incubating in the light incubator, with subculturing for 15 days of each generation. The final OD was read at the end of the tests for up to five generations that showed no significant variations among the generations, indicating good genetic stability of the mutants up to the 5th generation.

### Growth of wild and mutant strains

**Fig. 6:**
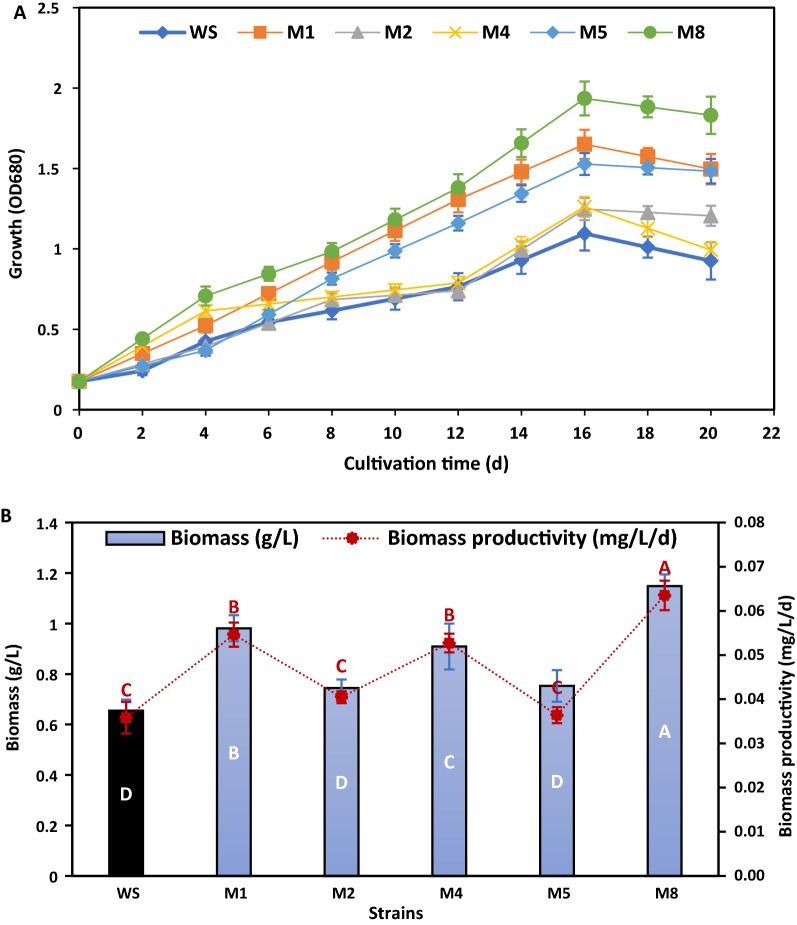
Growth performance of mutant and wild strains of *Parachlorella kessleri* in F/2 medium: **a** growth curve through 20 days of growth, **b** biomass, and biomass productivity at late exponential phase (16th day) (wild strain was colored black). Similar letters in each drawn series show insignificant differences at *p* < 0.05 using DMRT

The mutant and wild strains were grown in 350 mL of F/2 medium to study the growth parameters, including biomass (OD_680_ and dry weight) and biochemical constituents (pigments, protein, carbohydrate and lipid contents). The sigmoid growth curves of the wild and mutant strains are displayed in Fig. 6a. It can be seen that there were no significant differences between the growth stages at the early stage of cultivation, with the exponential growth phase starting on the 2nd day and continuing up to the 16th day for all strains, which then followed the stationary phase (Fig. 6a). Among the strains, M8 showed the highest growth with biomass of 1.14 g/L and productivity of 0.064 mg/L/day, which increased by 77.23% and 75.41%, respectively, compared to the wild strain. Strains M1 and M5 were in the second and third orders, respectively. In contrast, strains M2 and M4 showed no significant difference in growth and biomass productivity compared to the wild strain (Fig. 6b). Compared to the literature, improvement in biomass concentrations and productivity of the M8 strain were significantly higher. For example, compared to the original strain, the dry biomass and lipid productivity of an ARTP mutant of *Chlorella pyrenoidosa* II-H6 were reported to increase by 22% and 17%, respectively [16]. An ARTP mutant strain of *Desmodesmus* sp. earlier showed a 15% improvement in biomass production [32]. Likewise, biomass production by a mutant strain of *P. kessleri* (PK4) obtained from the heavy-ion beam irradiation was increased by 22% [33].

### Biochemical compositions

**Fig. 7:**
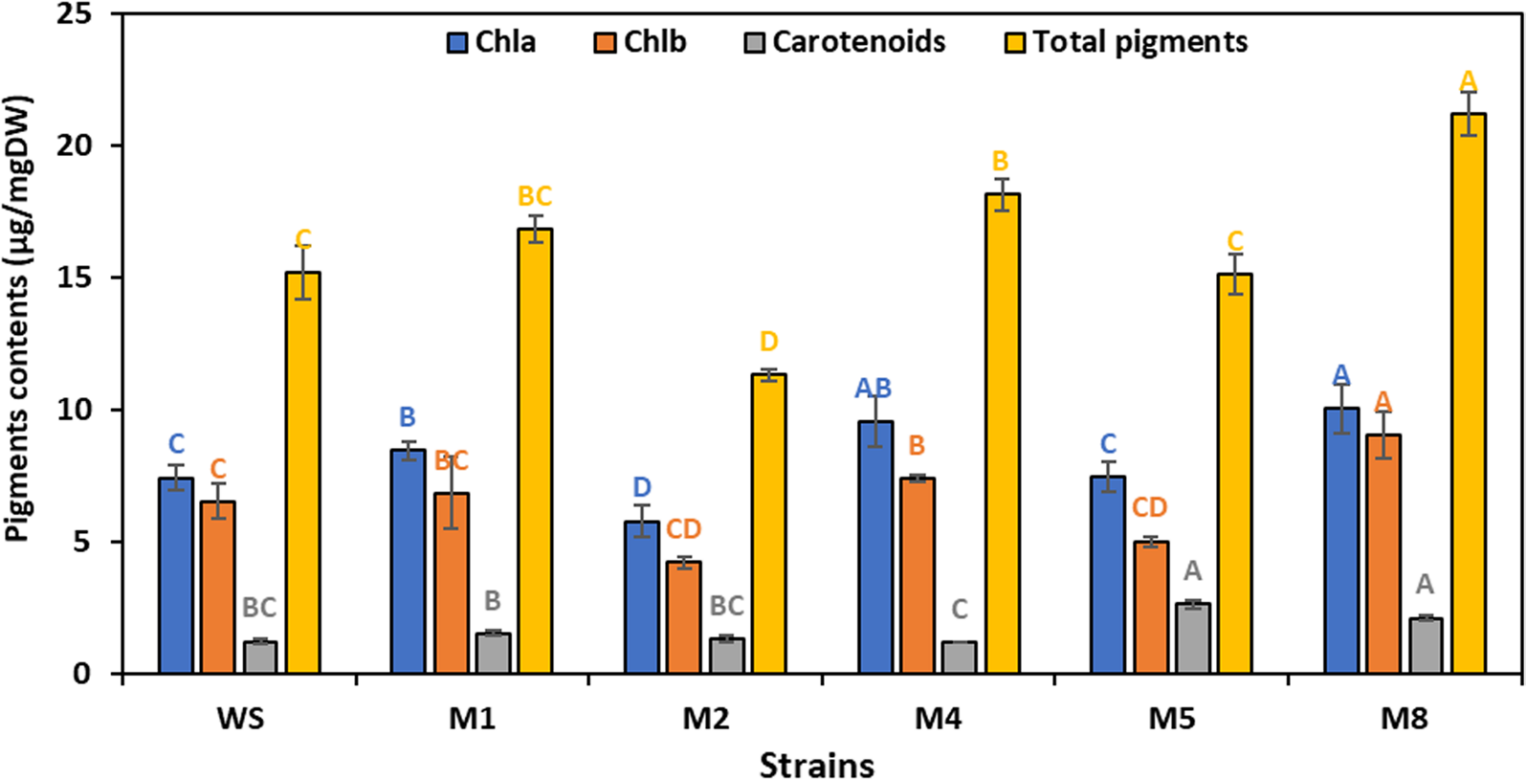
Chlorophyll *a*, chlorophyll *b*, carotenoids, and total pigment contents (µg/mgDW) of mutant and wild strains of *Parachlorella kessleri* at late exponential phase (16th day). Similar letters in each drawn series show insignificant differences at *p* < 0.05 using DMRT

The biochemical composition, including pigments, proteins, carbohydrates and lipids, of the mutant and wild strains were studied as shown in Fig. 7. At the late log phase (16th day), the maximum pigment content was found in M8 among the strains. The pigment content of the mutants varied significantly from the wild strain (*p* < 0.05), except for the pigment content of strain M5, which did not differ significantly from the pigment content of the wild strain (*p* > 0.05). It was observed that the pigment content of the strains positively correlated with biomass concentration and productivity (*r* = 0.83 and 0.82, *p* ≤ 0.05). Likewise, the maximum contents of Chl a and b were recorded in strains M8 (10.04 and 9.03 µg/mgDW, respectively), which were 1.35 and 1.38-fold higher than the wild strain. On the other hand, strain M5 showed the highest carotenoid content (2.63 µg/mgDW). These results were comparable with the findings of earlier studies that reported a ~ twofold increase in the total Chl of *Spirulina platensis* by applying ARTP [20, 34]. In contrast, the chlorophyll content and fluorescence of *Chlamydomonas reinhardtii* were reported to decrease significantly after ARTP treatment [19].

**Fig. 8:**
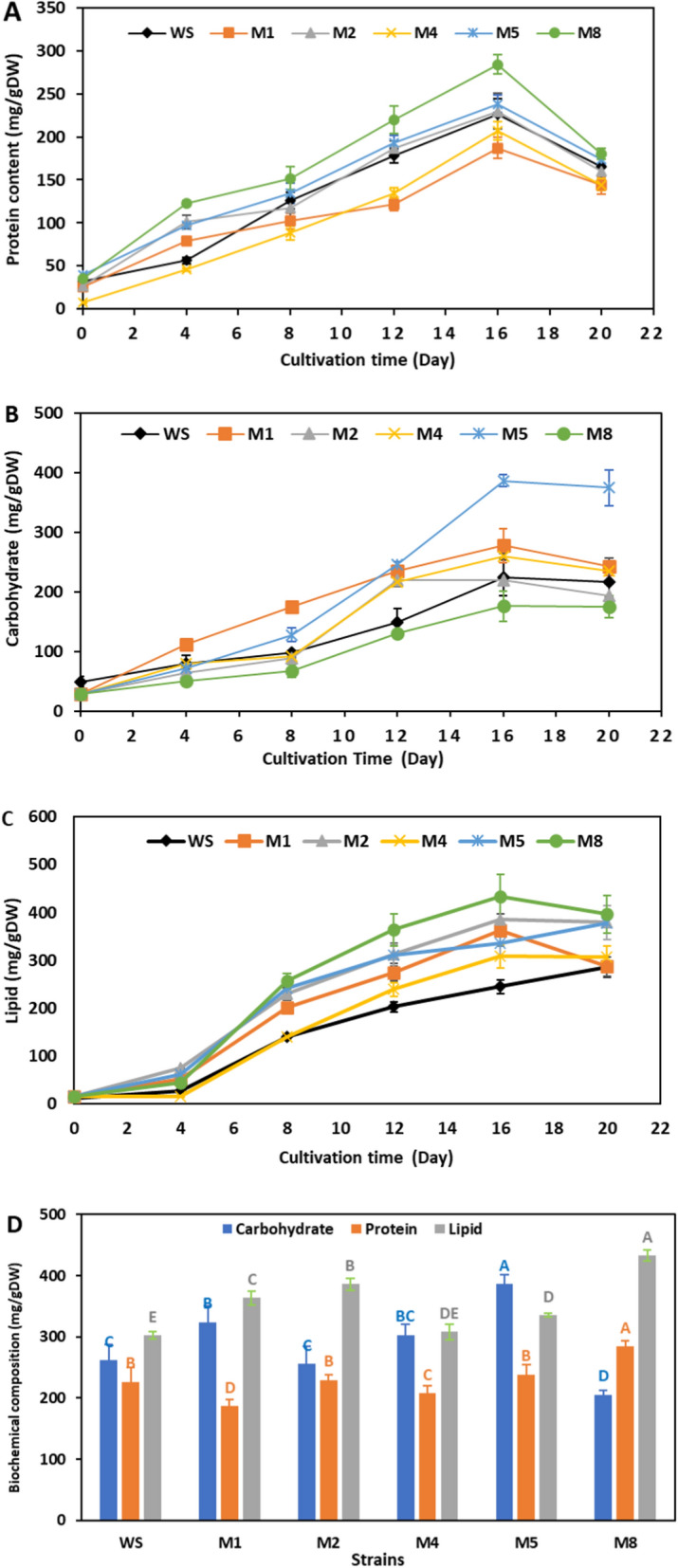
The biochemical composition (mg/gDW) of mutant and wild strains of *Parachlorella kessleri*. **a** Protein, **b** carbohydrate, **c** lipid during cultivation time, and **d** the highest yield of biochemical composition at late exponential phase (16th day)

Protein, carbohydrate and lipid of the wild and mutant strains were estimated every 4 days, which increased with an increase in biomass production throughout the initial period of cultivation and continued until 16th day (Fig. 8), which later dropped due to the algal age, light limitation in the dense culture, and nutrient exhaustion [35, 36]. Among the five mutants, M8 showed the highest protein content (284.2 mg/gDW, 28.42%DW), followed by M5 and M2, which increased by 25 and 5%, respectively, from the wild strain (226.6 mg/gDW, 22.66%DW), while strains M1 and M4 showed a decrease in protein content (Fig. 8d; Table 2).

**Table 2:** Protein, carbohydrate, and lipid contents (%DW) of the mutant and wild strains of *Parachlorella kessleri* at the late exponential phase (16th day)

Strains	Protein (%DW)	Carbohydrates (%DW)	Lipid (%DW)
WS	22.66 ± 1.68^b^	26.12 ± 1.82^c^	24.43 ± 1.71^f^
M1	18.72 ± 1.13^d^	32.41 ± 2.89^b^	36.32 ± 2.54^c^
M2	22.97 ± 2.05^b^	25.60 ± 1.56^c^	38.59 ± 2.70^b^
M4	20.74 ± 1.34^c^	30.29 ± 0.90^b^	30.81 ± 2.15^e^
M5	23.85 ± 1.35^b^	38.64 ± 2.21^a^	33.56 ± 2.35^d^
M8	28.42 ± 1.76^a^	20.57 ± 1.14^d^	43.29 ± 3.03^a^

Similar letters in each column represent insignificant differences at *p* < 0.05 using DMRT

The carbohydrate content significantly improved in the mutant strains over the original strain except in strains M2 and M8. The highest increase was recorded in strain M5 with 38.64% (386.44 mg/gDW), which was 1.47-fold greater compared to the wild strain (261.27 mg/gDW, 26.12%DW), followed by strains M1 and M4, respectively (Fig. 8d; Table 2). On the other hand, the lipid content showed the most effective constituent to the ARTP mutation. The lipid content was improved in all mutant strains, ranging from 26 to 77% compared to the wild strain. M8 strain showed the highest lipid content (432.93 mg/gDW, 43.29%DW) with an increase of 77% over the wild strain, followed by strains M2, M1, M5 and M4, respectively (Fig. 8d; Table 2).

The change in the content of cell components has been previously confirmed by ARTP mutagenesis [12, 20]. In the present work, the M8 strain showed low carbohydrate content but a significant pigment, protein and lipid accumulation. This finding was probably because ARTP mutation could alter the metabolic activities and storage components [37, 38]. Sun et al. [12] demonstrated that cellular components of *Desmodesmus* sp. were significantly altered by ARTP mutation wherein strain AT60-5, lipid and protein content were enhanced at the expense of carbohydrates. Despite lipid synthesis being more complicated than protein and carbohydrates, biomass and lipid accumulation mostly depend on the photosynthetic rate and transformations of other low-energy compounds such as proteins (16.7 kJ/g) and carbohydrates (15.7 kJ/g) [37, 39]. It was the main reason why M8 represented higher biomass and photosynthetic capacity. Current ARTP mutant *P. kessleri* showed a higher lipid content of 43.29% DW, while heavy-ion beams irradiated mutant *P. kessleri* improved lipid content to only 31% DW [33]. As a result, the dominant strains that benefited from the optimal ARTP radiation period had different characteristics, such as increased biomass and lipid storage. Lipid accumulation in M8 achieved the highest peak of 43.29%, while carbohydrates showed lower levels of 20.5%. Therefore, strain M8 was the mutant that demonstrated the highest degree of lipid accumulation and may need further investigation into large-scale and cost production to be suitable for biodiesel production.

### Triglyceride (TAG), lipid productivity and fatty acid content

**Fig. 9:**
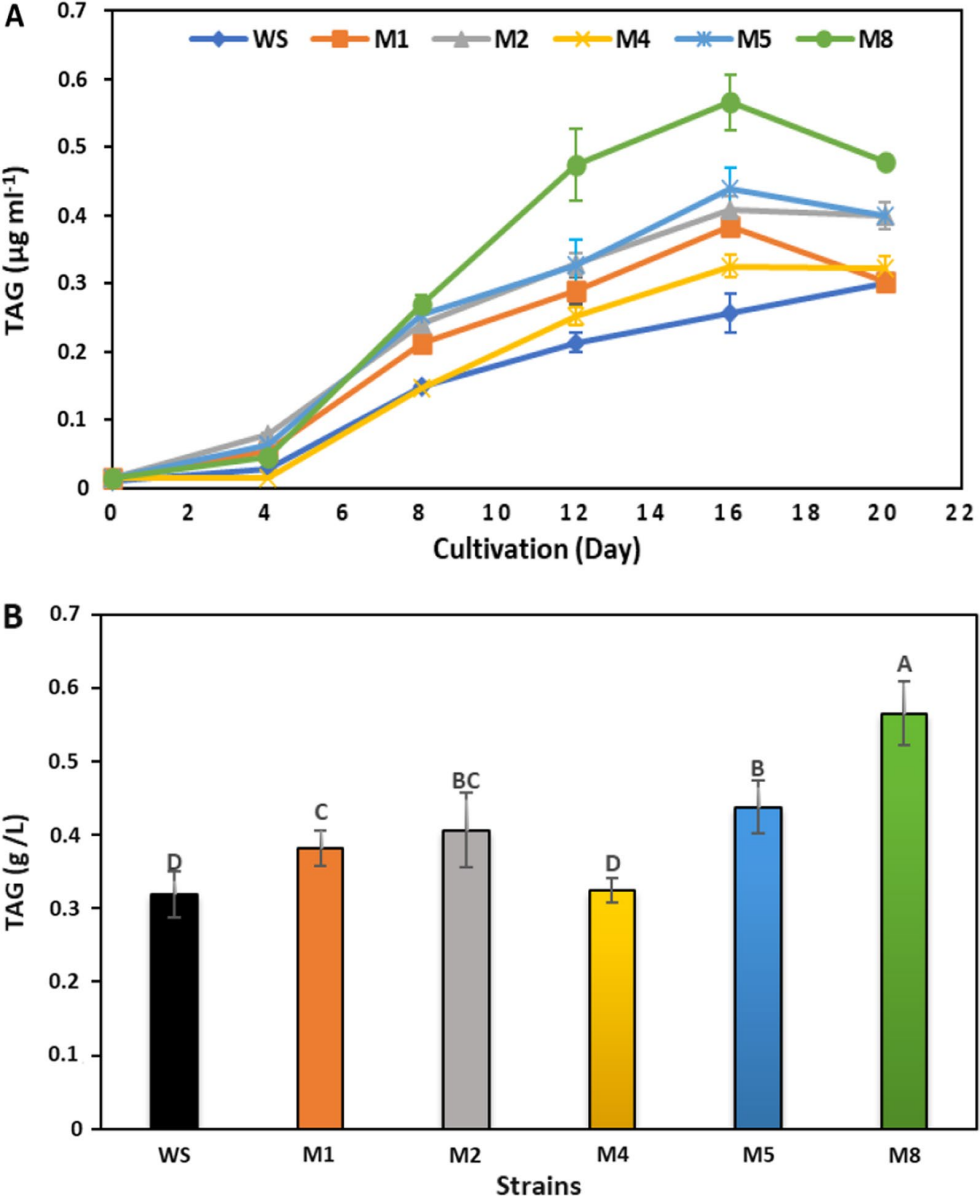
Triglyceride content (g/L) in the mutant and wild strains of *Parachlorella kessleri* during cultivation time (**a**) and the highest yield of triglyceride yield at late exponential phase (16th day) (**b**)*.* Different letters in each plotted series show significant differences at *p* < 0.05 using DMRT

Triglyceride (TAG) is the most common neutral lipid, accounting for more than 80% of total lipid [40]. TAG production is measured using the Nile red method, which provides estimates of lipid accumulation [12, 16]. TAG production was compared in order to obtain the best mutants with high lipid production for biodiesel production (Fig. 9). The amount of TAG accumulated by the mutants varied significantly (ANOVA, *p* < 0.05).

The results revealed that the TAG content increased in the mutants, which was almost stable after the 16th day in the mutants and original strain (Fig. 9a). There was a substantial improvement in TAG accumulation between the mutants (M1, M2, M5 and M8) and the wild strain (ANOVA, *p* < 0.05). The mutant of M8 showed the highest TAG accumulation than the other strains. TAG accumulation in M8 was 565.2 µg/mL, which was 1.77-folds higher than the wild strain (318 µg/mL). M5 strain was represented in second order with a TAG content of 437.96 µg/mL, while there were no significant variations between M4 strain and the wild strain (Fig. 9b). The mutated strains exhibited superior lipid production compared to the wild strain. A previous study reported that the ARTP mutants of *Desmodesmus* sp. showed higher TAG content than the wild strains [12, 32]. Multiple enzymes are involved in TAG biosynthesis and are controlled by gene regulation. Although TAG gene expression was not established in this study, higher relative TAG gene expression was confirmed in the high-lipid ARTP mutants of *Desmodesmus* sp. compared to the wild strains such as acetyl-coenzyme A carboxylase (accD), diacylglycerol acyltransferase (dgat7566), phospho- enol pyruvate (pepc6833), and malic enzyme (me6562) [12].

Despite increased TAG and lipid content being the most important component in identifying the optimal conditions for biodiesel production, lipid productivity and quality of the fatty acids (FA) profile determine whether the lipid is acceptable for biodiesel production. Microalgae can change the intracellular lipid production pathways to accumulate neutral lipids instead of structural lipids due to different stresses and mutations. In this context, lipid accumulation was previously confirmed in mutant microalgae [16, 41]. Another study showed that high-lipid accumulation in ARTP-mutated *Desmodesmus* sp. is associated with the upregulation of fatty acid synthases genes, including acetyl-CoA carboxylase (ACACA), beta-ketoacyl synthase II (fabF), 3-oxoacyl-[acp] reductase (fabG) and long-chain acyl-CoA synthetase (ACSL) [32]. In this study, the maximum lipid productivity of 20.19 mg/L/day was observed in the M8 strain with an increase of 79% than the wild strain, which was higher than that was detected in ARTP mutant *Chlorella pyrenoidosa* (II-H6) that was enhanced by 16.85% compared to the wild strains [16] and ARTP mutant *Desmodesmus* sp. (70%) [12].

**Fig. 10:**
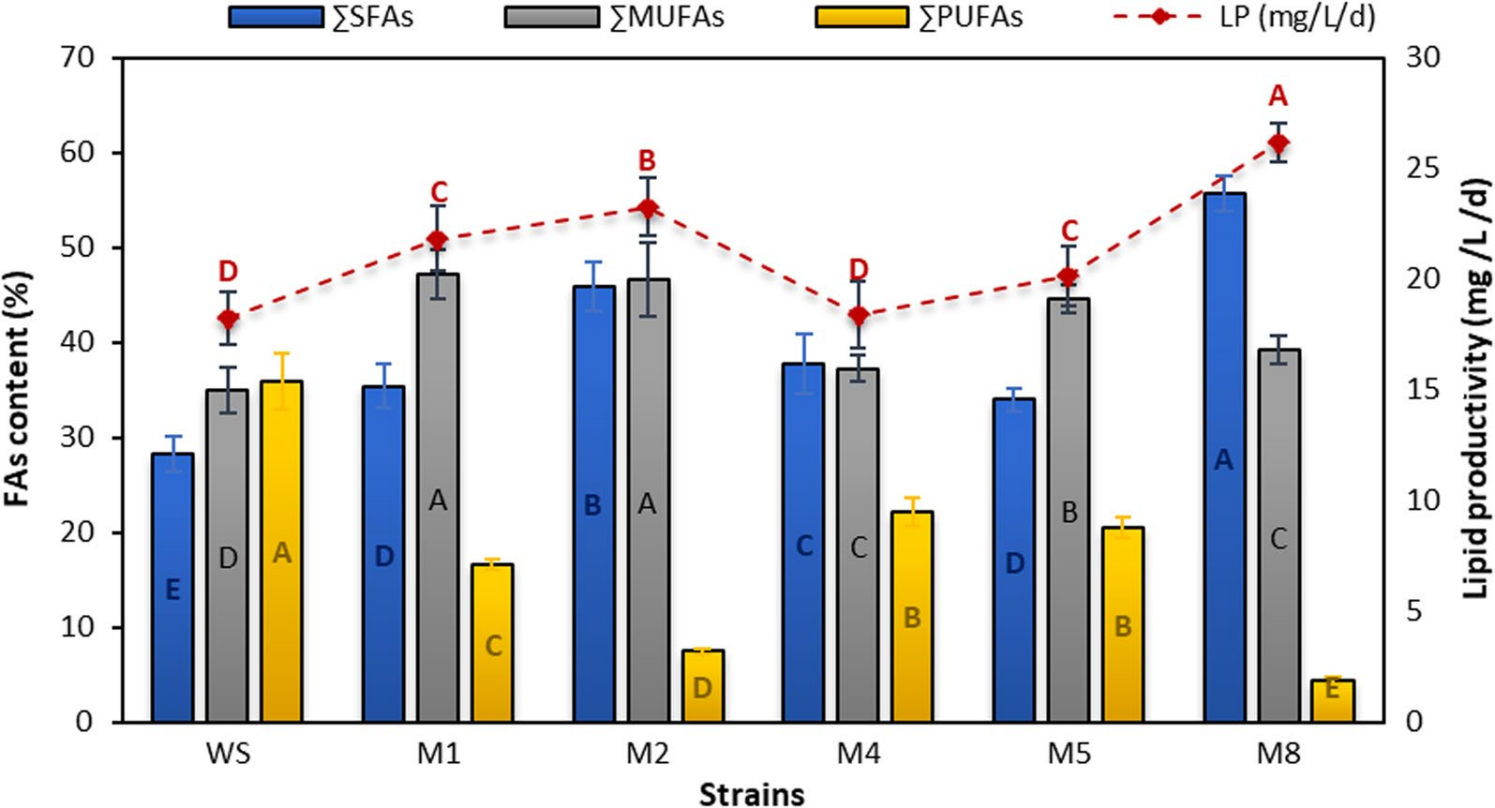
Fatty acid contents (%) and lipid productivity (mg/L/day) in the mutant and wild strains of *Parachlorella kessleri*. Similar letters in each drawn series show insignificant differences at *p* < 0.05 using DMRT

Fatty acid fractions were also varied in different strains; the highest saturated fatty acid (SFA, 56.7%) was recorded in the highest lipid productive strain of M8, corresponding to about twofold than those produced by the wild strain. The M2 strain showed the highest monounsaturated fatty acid (MUFA, 46.04%). On the other hand, the wild strain exhibited the maximum polyunsaturated fatty acid (PUFA, 35.9%; Fig. 10) that was not preferred in biodiesel production. Interestingly, the SFA of all mutant strains improved compared to the wild strain, while the PUFA content decreased compared to the wild strain. Under the above conditions, the mutant strains will benefit as biodiesel producers [10, 42]. On the other hand, MUFA content does not differ significantly between the wild and mutant strains. Algal lipids showed higher SFA levels than the usual biodiesel feedstocks of soybean and canola oil, making them preferable for biodiesel synthesis [43].

### Fatty acids profile

Mutants and original *P. kessleri* cells demonstrated different values of the proportion of individual fatty acids (FA). 20 FA were found in the six strains of *P. kessleri* within the carbon chain length of C8–C26. However, the predominated FA were palmitic acid (C16:0), stearic acid (C18:0), oleic acid (18:1), linoleic acid (18:2) (Table 3; Additional file 1: Fig. S1), which have previously been recorded in different biodiesel recommended [44] and other microalgae species [6, 45]. SFA comprising palmitic acid (C16:0) and stearic acid (C18:0) showed the major proportion in *P. kessleri,* which varied in different mutant strains. The highest content of these FA was recorded in the M8 strain. Our results support a former study documenting that *P. kessleri* strains had increased palmitic and stearic acid content [23, 46]. The current study discovered that the ARTP mutation significantly impacted the compositions of fatty acids and the degree of saturation.

**Table 3:** Relative proportion (%) of the fatty acid profile in the mutant strains and wild strains of *Parachlorella kessleri* at the late exponential phase (16th day)

FA	WS	M1	M2	M4	M5	M8
C8:0	–	–	–	–	–	0.55
C10:0	1.06	–	–	–	–	1.14
C12:0	–	0.17	0.72	–	0.10	2.32
C14:0	0.18	0.26	0.23	0.23	0.16	0.44
C16:0	22.00	27.83	36.16	30.81	25.86	39.52
C18:0	3.06	5.20	8.09	4.27	5.48	9.10
C20:0	0.46	1.68	0.38	2.40	2.01	2.39
C22:0	1.09	0.12	0.18	–	0.12	0.23
C24:0	0.44	0.13	0.14	–	0.15	–
C26:0	–	–	–	–	0.05	–
C16:1 (ω7)	–	1.49	1.25	2.18	0.70	0.49
C18:1 (ω7)	4.46	2.06	–	–	2.85	2.73
C18:1	–	–	2.64	–	–	–
C18:1 (ω9)	30.51	43.31	42.57	35.07	40.87	35.39
C20:1 (ω9)	–	0.26	0.10	–	0.23	0.58
C16:2	1.67	1.28	0.38	2.61	1.05	–
C18:2(ω6)	25.28	13.66	6.91	16.61	18.03	4.40
C16:3	–	1.65	–	–	1.39	–
C18:3 (ω3)	8.95	–	0.21	4.94	–	–
C22:4 (ω6)	–	–	0.15	–	–	–

–, means not detected

Regarding the MUFA, oleic acid was the dominant FA representing 30.51–43.31% and the highest content represented in M1. Linoleic acid was the dominant FA in PUFA, and the original strain showed the highest contents of 25.28%. In the mutant strains, more C16 (22–73%) and less C18 accumulated than in the original one. The present results revealed that mutant strains of *P. kessleri* accumulated mainly C16 and C18, accounting for >92% of total FA. Furthermore, no polyunsaturated fatty acids with ≥4 double bonds were present in the mutation stains (except M2 strain) and showed low unsaturation levels of FA. Such composition and structure of FA are recommended for biodiesel production. Accordingly, these mutant strains were used to estimate their biodiesel quality.

### Biodiesel properties

The properties of biodiesel were calculated and compared to those recommended by American [47] and European [48] international standards (Table 4). All the tested strains met the acceptable ranges of international standards for cetane number, kinematic viscosity, specific gravity, 18:3 FA content, and FA with four or more double bonds (Db ≥ 4).

**Table 4:** The estimated properties of biodiesel derived from the mutant strains and wild strains of *Parachlorella kessleri* in comparison with the international standards

Biodiesel parameters	WS	M1	M2	M4	M5	M8	ASTM D6751	EN 14214
ADU	1.47	1.23	1.07	1.20	1.27	0.87	–	–
KV	4.28	4.43	4.53	4.45	4.40	4.66	1.9–6.0	3.5–5
CN	53.05	54.65	55.74	54.85	54.38	57.09	≥ 47	≥ 51
IV	122.29	104.48	92.27	102.26	107.50	77.19	–	≤ 120
Cp	0.32	3.51	5.71	3.91	2.97	8.41	–	> 4
Density	0.88	0.88	0.88	0.88	0.88	0.88	0.85–0.90	0.86–0.90
HHV	41.13	40.71	40.42	40.65	40.78	40.06	–	–
Db ≥ 4	8.95	0.00	0.22	4.95	0.00	0.00	≤ 1	0
C18:3	8.95	0	0.22	4.94	0	0	≤ 12	≤ 12
LCSF	6.69	7.40	8.59	7.61	7.62	11.23	–	–
CFPP	4.53	6.78	10.49	7.44	7.46	18.82	–	–

–, means not detected

The highest fatty acids unsaturation degree was recorded in the original strain of 1.25 ∇/mol, which was reduced significantly in the mutant strains, and strain M5 showed the lowest degree of 0.48 ∇/mol. It is widely known that the low unsaturation degree is a key factor in establishing the qualities of biodiesel. Cetane number (CN) implies the fuel's ignition quality in the engines, and a greater value of CN for conventional diesel alternatives is advantageous. The biodiesel of strain M5 had the greatest CN of 57.09, compared to 53.05 of the original strain, allowing the engine to start faster and quieter, showing improved ignition efficacy and low NO_*x*_ releases [49, 50], as well as better oxidative stability.

Although all strains met the iodine value (IV) allowed by EN 14214 standards of ≤ 120 g *I*_2_/100 g oil, which is not represented in most biodiesel feedstock such as soybean or sunflower [51]. The lower IV recorded in the mutant strains indicates a lower saturation level and high stability against oxidation [52]. Our results were consistent with different biodiesels derived from different green microalgae [11, 53].

Kinematic viscosity (KV) and specific gravity (ρ) were also coordinated with international standards [47, 48], with no significant differences among strains. Cloud point indicates the temperature at which biodiesel begins thickening and cannot flow accordingly the CP in the American standard is more than 4 °C. All the mutant strain was within the range, while the original one was out of the range. In general, the cetane number and viscosity rise with increasing the saturation degree of FA, while the oxidative stability and cold flow values are reduced [54].

A higher heating value (HHV) is the amount of heat released by the full combustion of a unit quantity of fuel. According to prior investigations, the HHV values found in this study were acceptable [55–57], and it concurs with earlier results for *P. kessleri* (39 and 40 MJ/kg) [30]. The highest long-chain saturated factor (LCSF) and the highest biodiesel quality [57, 58] were also recorded in strain M8. Cold filter plugging points (CFPP) of the FAMEs of all strains were found to be suitable for use in moderate-temperature regions [59]. Overall, mutant strain M8 of *P. kessleri* generated biodiesel that matched international standards and might compete with fossil diesel as industrial biodiesel in the future.

## Conclusion

This study demonstrated that ARTP-based mutagenesis is both efficient and simplistic. Screening of mutants for optimal features is also necessary to further evaluate the developed mutant strains. According to the distinguishing qualities of particular growth rate, optical density, and lipid content, the best M8 strain was obtained from 15 colonies. Selective mutant strains were cultivated for five generations that showed stable characteristics. High lipid productivity, saturated fatty acids, and C16–18 FA concentration were found in mutant strain M8. Furthermore, compared to the wild strain, the FAMEs profile and biodiesel characteristics of the M8 strain were greatly improved, indicating its potential as a biodiesel feedstock. Microalgae mutation using ARTP to enhance lipid productivity, fatty acid composition, and biodiesel quality could be used in the production of biodiesel. However, further fuel quality scale-up and validation testing are recommended.

## Material and methods

### Microorganisms and culture conditions

A coccoid green alga species was isolated from the Yellow Sea in China (34° 47′ 49.5″ N 119° 16′ 46.8″ E) and purified using the subculturing approach in F/2 medium supplemented with 30 g/L of commercially available marine salts [60]. Stock culture was maintained in F/2 medium with the 24 h fluorescent light of 300 μmol/m^2^/s at 25 ± 3 °C. The coccoid microalga was morphologically recognized as Chlorellales species. Standard microscopic examination (Olympus BX51 light microscope, Tokyo, Japan) and re-subculturing procedures were used to establish the axenic culture of isolated microalgae. The isolated microalgae were preserved in agar slants of F/2 medium at 4 °C.

### Molecular identification of the isolate using 18S rDNA sequencing

The total genomic DNA of the isolated microalga was extracted and purified using the DNA extraction and purification kit, following the manufacturer's instructions [35]. Algal DNA was amplified with Super Pfx DNA Polymerase (Cwbiotech, Beijing, China) using the universal 18S rRNA primers, CdmF (5′-GTCAGAGGTGAAATTCTTGGATTTA-3′) and CdmR (5′-AGGGCAGGGACGTAATCAACG-3′) [61]. PCR thermal cycling consists of 95 °C for 5 min, followed by 34 cycles of 1 min at 96 °C, 30 s at 60 °C, 1 min at 74 °C, and a final extension at 74 °C for 10 min using the thermal cycler (Bio-Rad Laboratories, USA). The PCR products were observed via agarose gel electrophoresis (1% w/v) with Sybr Safe DNA Gel Stain. The Fast Pure Gel DNA Extraction Mini Kit was used to purify DNA fragments corresponding to the 18S rRNA gene (Vazyme, Nanjing, China) and later sequenced at the Hongxun Technology (Suzhou, China). The 18S rDNA sequences were aligned with the matched sequences from the NCBI GenBank using nBLAST. A phylogeny was generated using the maximum parsimony (MP) [62] and neighbor-joining (NJ) algorithm [63] based on the parameter distance (PD) [64] using the MEGA-X software. The bootstrap test (1000 replicates) was done for NJ-PD in MEGA-X, and values ≥70 are stated in the phylogenetic tree.

### Algal biomass assay

The alga was grown in three replicates of static cultures in 200 mL of F/2 medium using a 500 mL Erlenmeyer flasks at the same optical density (OD_680_) of ~ 0.1 (with the initial inoculums of 25.0 ± 0.5 mg DW) using inoculant in log phase for 20 days under the 24 h fluorescent light of 300 μmol/m^2^/s at 25 ± 3 °C and manually shacked 3 times daily to avoid sticking of biomass to the bottom of the tube. Cell growth was detected every couple of days by aliquot 2 mL of culture for estimating the OD_680_. Dry weight (DW) was determined using a calibration curve correlating to OD_680_ values, *R*^2^ = 0.98. Growth rate µ (day^−1^) was estimated according to Eq. (1) [10]:

1 $$\mu = \frac{{\ln \,{\text{DW}}_{t} - \ln \,{\text{DW}}_{0} }}{t},$$

where DW_*t*_ and DW_0_ are the final and initial dry weight (g/L), and t represents the culture time.

### ARTP mutagenesis and screening

Under aseptic conditions, 50 µL of 10% glycerol (a protective agent) was mixed with 50 µL of algal culture (0.1 OD_680_) in the exponential phase of growth in F/2 medium, where cells possessed high vitality and were the best for mutation process [16]. ARTP was accomplished in the ARTP mutagenesis mutating machine (Wuxi Yuanqing Tianmu Biological Technology Co., Ltd., Wuxi, China). In brief, 10 μL of algal suspension was deposited on a stainless-steel slide (12 mm in diameter) and subjected to ARTP. The ARTP machine was adjusted at radio-frequency power input 100 W, the distance between slide and plasma emitter jet 2 mm, helium gas flow rate of 10 L/min, and exposure time differed from 10 to 60 s. The strain without exposure to ARTP was used as a control. After ARTP treatment, the contents of the stainless-steel plates were transferred to 1 mL of liquid F/2 medium and vortexed for 30 s to resuspend the algal cells. Then, 100 μL of resuspending algal cells were streaked on regular F/2 medium agar plates and incubated upside down on the light incubator at 25 ± 2 °C for 7 days to obtain desired mutants. The viable strains were picked and spread on the new agar plates. After the incubation period, the colonies of the mutant samples (*T*) and the control (*C*) were counted, and the mortality rate (%) was calculated according to Eq. (2). The single growing colonies were picked with a sterilized needle and then cultivated in 50 mL of fresh F/2 medium and incubated under light conditions. Algal growth, growth rate and cellular composition were estimated:

2 $${\text{Mortality rate }}\left( {\text{\% }} \right) = 1 - \left( {{\raise0.7ex\hbox{$T$} \!\mathord{\left/ {\vphantom {T C}}\right.\kern-\nulldelimiterspace} \!\lower0.7ex\hbox{$C$}}} \right) \times 100.$$

### Determination of cellular composition

To determine the chemical composition, a 1-L flask containing 600 mL of F/2 medium was cultivated with the same initial inoculums of 25.0 ± 0.5 mg DW for all strains and incubated under the same condition mentioned above in three replicates. A volume of 3 mL of algal culture at the exponential growth was centrifuged at 4000×*g* for 8 min; then the algal cells were extracted with 90% methanol in a 55 ℃ water bath for 30 min. Chlorophyll a, b and carotenoids were determined spectrophotometrically at 644, 663 and 452 nm [65] according to Eqs. (3) and (4):

3 $${\text{Chl}} a = 10.3 \times A_{663} - 0.913 \times A_{644} ,$$

4 $${\text{Chl}}\, b = 1907 \times A_{644} - 3.87 \times A_{663} ,$$

5 $${\text{Carotenoids}} = 4.2 \times A_{452} - \left( {0.0264 \times {\text{Chl}} \,a + 0.426 \times {\text{Chl}}\, b} \right).$$

For carbohydrate and protein estimation, 10 mL of algal culture was collected every 4 days and centrifuged at 4000×*g* for 8 min; then the algal pellet was hydrolyzed by 10 mL of 1 N NaOH in boiling water for 2 h. Total soluble carbohydrates and proteins in the NaOH hydrolysate were estimated spectrophotometrically at 490 nm by the phenol–sulfuric acid method [66] modified by [35] and the Bradford method at 490 nm [67], respectively.

The lipid content was estimated by a sulfo-phospho-vanillin (SPV) assay [68]. Two milliliters of culture was centrifuged at 8000×*g*, the supernatant was removed, and the algal cells were homogenized with 2 mL conc. H_2_SO_4_. The sample was then digested for 10 min in a boiling water bath (100 ℃) and followed by cooling in ice bath for 5 min. Thereafter, 5 mL of freshly made phospho-vanillin mixture was added to the digested sample and incubated for 15 min at 37 ℃ in a rotary shaker incubator. A noticeable pink color was developed at the end of the reaction. The absorbance of the resulting sample was determined at 530 nm spectrophotometry against sunflower oil standard curves. The SPV method is simpler to use and less time-consuming than the gravimetric method, and it requires significantly less biomass. Furthermore, the organic solvent method extracts non-lipid components as well as lipid components, resulting in inaccurate measurements of lipid content in cells compared to SPV methods [69].

Triglycerides (TAG) were determined by the Nile red method [70, 71]. Algal cultures were treated with Nile red dye (5 μg/mL in acetone) directly in 1:100 v/v (Nile red:algal culture) for 20 min in the dark. The fluorescence of the stained cells was detected at 490 nm for excitation and 585 nm for emission by using a Tecan Infinite 200PRO microplate reader (Tecan, Männedorf, Switzerland). Each measurement was repeated three times.

### Estimates of productivities

Biomass (BP) and lipid productivities (LP) were estimated according to [9], as shown in Eqs. (6) and (7), respectively:

6 $${\text{BP}} = \frac{{{\text{DW}}_{{{\text{EL}}}} - {\text{DW}}_{{{\text{LL}}}} }}{\Delta t},$$

7 $${\text{LP}} = \frac{{{\text{LC}}_{{{\text{EL}}}} - {\text{LC}}_{{{\text{LL}}}} }}{\Delta t},$$

where DW_EL_ and DW_LL_ are dry biomass (g/L) in the early and late logarithmic growth phases, respectively. *L*_EL_ and *L*_LL_ were the lipid content (mg/L) in early and late log phases, and Δ*t* is the difference in cultivation time.

### Analysis of the fatty acid composition

Approximately 250 mL of algal cells was cultivated for 16 days till the end of the exponential growth phase, then centrifuged at 4000×*g* for 8 min. The algae pellet (100 mg fresh weight) was mixed with the chloroform–methanol mixture (2:1) (1:10 w/v) and 1 mL of 5% NaCl. The lipid extracts were dried in a dry oven at 80 °C to constant weight [72].

Once lipid extraction, lipid content was transformed into fatty acid methylated ethers (FAMEs) using sodium methoxide [27]. The FAMEs composition was identified using an internal standard (F.A.M.E. Mix, C4-C24 from Sigma-Aldrich, SUPELCO SKU: 18919-1AMP) or by mass spectroscopy using gas chromatography–mass spectrometry (Perkin Elmer model: Clarus 580/560S, Norwalk, USA) with a packing column Elite-5MS (30 m × 0.25 mm × 0.25um). The mass spectrometric detector was operated in scanning from 50 to 620 Da. The temperature was initiated at 60 °C for 6 min, then continued to increase to 140 °C at a rate of 5 °C/min and held for 2 min. Then the temperature was elevated to 280 °C gradually at a rate of 5 °C/min and held for 5 min. One microliter of FAMEs was injected in a split ratio of 1:20. The solvent delay was 5 min. Helium gas was utilized at a flow rate of 1 mL/min, with the injector and detector temperatures set to 280 °C. The integrated peak areas were calculated after normalization to obtain the relative percentage of the FAMEs profile.

### Biodiesel properties

The quality of the produced biodiesel was assessed by estimating various physicochemical properties of FAMEs theoretically. Average Degree of Unsaturation (ADU%), Kinematic viscosity (*υ*_*i*_, mm^2^/s), density (*ρ*), Cetane Number (CN), cloud point (CP), Cetane Number (CN), Iodine Value (IV, g I_2_.100/g oil), Long Chain Saturation Factor (LCSF, wt%), Higher Heating Value (HHV), Long Chain Saturation Factor (LCSF, wt%) and Cold Filter Plugging Point (CFPP, °C) were calculated according to [73–77]. The obtained values of different parameters (according to Eqs. 8–16) were compared with the respective standard values discussed in the American Society for 2 Testing and Materials International (ASTM D-6751-02) and European biodiesel standard (EN-14214) to evaluate the properties of biodiesel to be produced from the biomass of *M. reisseri* grown in wastewater:

8 $${\text{ADU}} = \, \sum N \times Mf,$$

where *N* and *Mf* are the number of C=C bonds and mass fraction of each FAME.

9 $$\upsilon_{{\text{i}}} = {-}0.{6313} \times {\text{ADU}} + {5}.{2}0{65,}$$

10 $$\rho \, = \, 0.00{55} \times {\text{ADU}} + 0.{8726,}$$

11 $${\text{CP }} = {-}{3}.{356} \times {\text{ADU}} + {19}.{994,}$$

12 $${\text{CN }} = {-}{6}.{6684} \times {\text{ADU}} + {62}.{876,}$$

13 $${\text{IV}}\, = \,{74}.{373}\, \times \,{\text{ADU}}\, + \,{12}.{71,}$$

14 $${\text{HHV}}\, = \,{1}.{76}0{1}\, \times \,{\text{ADU}}\, + \,{38}.{534,}$$

15 $${\text{LCSF}}\, = \,\left( {0.{1}\, \times \,{\text{C16}}:0} \right)\, + \,\left( {0.{5}\, \times \,{\text{C18}}:0} \right)\, + \,\left( {{1}\, \times \,{\text{C2}}0:0} \right)\, + \,\left( {{2}\, \times \,{\text{C24}}:0} \right),$$

16 $${\text{CFPP}}\, = \,{3}.{1417}\, \times \,{\text{LCSF}}{-}{16}.{477,}$$

where C16:0, C18:0, C20:0, C22:0 and C24:0 are the proportion of the corresponding FAME.

### Statistical analysis

All experiments were accomplished in triplicate, and the results are expressed as the mean ± SD. One-way analysis of variance (ANOVA) was applied to evaluate the statistical significance among groups of only one independent variable. Duncan multiple range test (DMRT) was used for multiple comparisons among averages from significant ANOVA tests. Spearman correlation was used to determine the relationship between various growth parameters. Data analyses were performed using Statistical Package for Social Sciences (SPSS) statistics software version 23 (IBM, USA) at a probability level of *p* ≤ 0.05.

## Supplementary Information

**Additional file 1.** Gas chromatogram of total fatty acids of *Parachlorella kessleri*, (A) wild strain, (B) M1, (C) M2, (D) M4, (E) M5 and (F) M8. Arrows show some characteristic fatty acids

## Data Availability

Not applicable.
