# Supplementary material for: Enhancing biomass and lipid productivity of a green microalga Parachlorella kessleri for biodiesel production using rapid mutation of atmospheric and room temperature plasma
Source: Biotechnol Biofuels Bioprod. 2022 Nov 13;15:122. doi: 10.1186/s13068-022-02220-z (PMC9655907; doi:10.1186/s13068-022-02220-z)
Supplement: Supplementary file 1 — Additional file 1. Gas chromatogram of total fatty acids of Parachlorella kessleri, (A) wild strain, (B) M1, (C) M2, (D) M4, (E) M5 and (F) M8. Arrows show some characteristic fatty acids [file 13068_2022_2220_MOESM1_ESM.pdf]

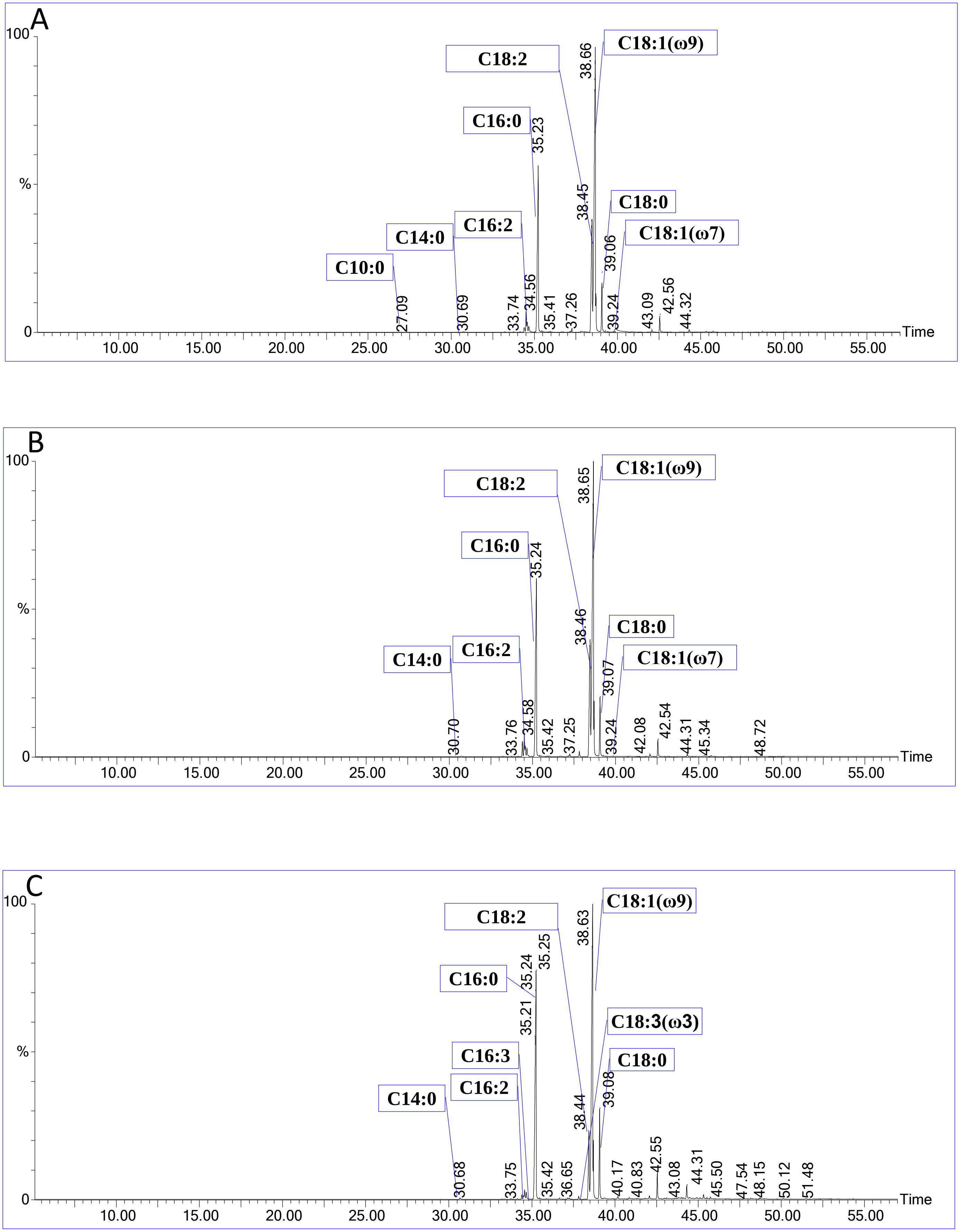
A
100
%
0
Time
C18:2
C18:1(ω9)
C16:0
C18:0
C16:2
C14:0
C18:1(ω7)
C10:0
38.66
35.23
38.45
39.06
27.09
30.69
33.74
34.56
35.41
37.26
39.24
43.09
42.56
44.32
10.00
15.00
20.00
25.00
30.00
35.00
40.00
45.00
50.00
55.00
B
100
%
0
Time
C18:2
C18:1(ω9)
C16:0
C18:0
C16:2
C14:0
C18:1(ω7)
38.65
35.24
38.46
39.07
30.70
33.76
34.58
35.42
37.25
39.24
42.08
42.54
44.31
45.34
48.72
10.00
15.00
20.00
25.00
30.00
35.00
40.00
45.00
50.00
55.00
C
100
%
0
Time
C18:2
C18:1(ω9)
C16:0
C18:3(ω3)
C16:3
C18:0
C16:2
C14:0
38.63
35.25
35.24
35.21
38.44
39.08
30.68
33.75
35.42
36.65
40.17
40.83
42.55
43.08
44.31
45.50
47.54
48.15
50.12
51.48
10.00
15.00
20.00
25.00
30.00
35.00
40.00
45.00
50.00
55.00

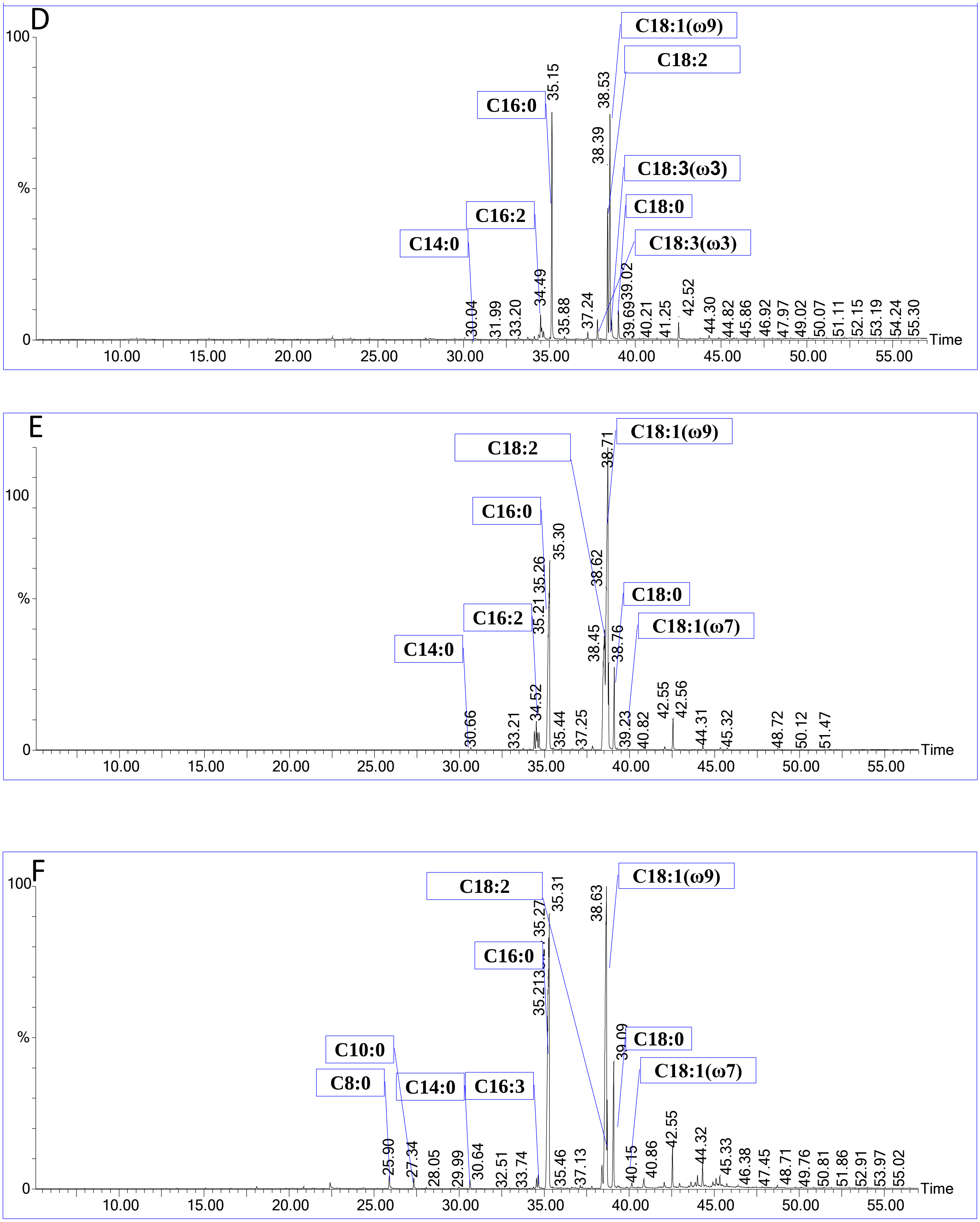

Fig. S1. Gas chromatogram of total fatty acids of *Parachlorella kessleri* , (A) Wild strain, (B) M1, (C) M2, (D) M4, (E) M5 and (F) M8, Arrows show some characteristic fatty acids.
